# A systematic review of the burden and risk factors of sudden infant death syndrome (SIDS) in Africa

**DOI:** 10.7189/jogh.11.04075

**Published:** 2021-12-25

**Authors:** Godwin K Osei-Poku, Sanya Thomas, Lawrence Mwananyanda, Rotem Lapidot, Patricia A Elliott, William B Macleod, Somwe Wa Somwe, Christopher J Gill

**Affiliations:** 1 Boston University School of Public Health, Department of Global Health, Boston, Massachusetts, USA; 2 Right to Care – Zambia, Lusaka, Zambia; 3 Boston University School of Medicine, Department of Pediatrics, Boston, Massachusetts, USA; 4 Boston Medical Center, Division of Pediatric Infectious Diseases, Department of Pediatrics, Boston, Massachusetts, USA; 5 Boston University School of Public Health, Department of Community Health, Boston, Massachusetts, USA; 6 University of Zambia, School of Medicine, Department of Pediatrics, Lusaka, Zambia

## Abstract

**Background:**

While sudden infant death syndrome (SIDS) has long been recognized as a leading preventable cause of infant mortality in high-income countries, little is known about the burden of SIDS in Africa. To address this knowledge gap, we conducted the first systematic review of SIDS-related publications in Africa. Our objective was to assess the prevalence of SIDS and its risk factors in Africa.

**Methods:**

We systematically searched PubMed, Embase, Web of Science, Cochrane, and Google Scholar to identify studies published until December 26, 2020. Review authors screened titles and abstracts, and selected articles independently for full-text review. Risk of bias was assessed using the Newcastle Ottawa Scale (NOS) or a modification. Data on the proportion of infants who died of SIDS and reported prevalence of any risk factors were extracted using customized data extraction forms in Covidence.

**Results:**

Our analysis rested on 32 peer-reviewed articles. Nine studies presented prevalence estimates on bedsharing and prone sleeping, suggesting near-universal bedsharing of infants with parents (range, 60 to 91.8%) and frequent use of the prone sleeping position (range, 26.7 to 63.8%). Eleven studies reported on the prevalence of SIDS, suggesting high rates of SIDS in Africa. The prevalence of SIDS ranged from 3.7 per 1000 live births in South Africa, 2.5 per 1000 live births in Niger, and 0.2 per 1000 live births in Zimbabwe. SIDS and other sudden infant deaths accounted for between 2.5 to 21% of infant deaths in South Africa and 11.3% in Zambia.

**Conclusions:**

Africa may have the highest global rate of SIDS with a high burden of associated risk factors. However, majority of the studies were from South Africa which limits generalizability of our findings to the entire continent. There is an urgent need for higher quality studies outside of South Africa to fill this knowledge gap.

**Protocol registration:**

Prospero Registration Number: CRD42021257261

In wealthier countries, sudden infant death syndrome (SIDS), a subset of sudden unexpected infant death (SUID), is recognized as a leading preventable cause of infant mortality. According to the US Centers for Disease Control and Prevention (CDC), SIDS was the leading cause of post-neonatal mortality and the fourth leading cause of infant mortality in the United States in 2017 [1]. SIDS is “the sudden and unexpected death of an infant under 12 months of age that remains unexplained after a review of the clinical history, complete autopsy and death scene investigation, with the onset of the fatal episode occurring during sleep” [2,3]. Nearly 90 percent of SIDS cases occur between birth and six months of age with a peak incidence around two to four months [4,5]. And while the pathobiology of SIDS may involve genetic or developmental factors, a key event in many SIDS cases is some form of respiratory stress, culminating in accidental suffocation. This is a critical point to note since the interventions that have successfully reduced SIDS deaths in the US and similar settings have largely focused on changes in infant sleeping conditions, most notably having infants sleep on their backs.

Sleeping in the prone or side position and bedsharing are recognized as major risk factors of SIDS [6]. The risk of re-breathing expired gases is increased in the prone or side sleeping position leading to hypoxia or hypercapnia [7]. Bedsharing is also important in suffocation deaths due to being accidentally rolled on by a sleeping adult. Infections and genetic polymorphisms have also been suggested in the etiology of SIDS [8,9]. Since symptoms of infectious diseases can be subtle in infants and hence not recognized ante-mortem, much attention has focused on the post-mortem identification of infectious pathogens. Multiple SIDS deaths in one family may also suggest a genetic link in the etiology of SIDS [10]. Other risk factors include little or no prenatal care, maternal age less than 20 years, prematurity or low birth weight, and maternal use of alcohol or smoking during pregnancy [11-14].

In sub-Saharan Africa, the contribution of SIDS to infant mortality has not been well understood. Very few studies have tried to estimate the incidence or prevalence of SIDS in Africa [15], and even fewer have studied the risk factors of SIDS [16]. Given the paucity of published studies on SIDS/SUID in Africa, this cause of child mortality has not been viewed as a high priority and has largely been ignored. And yet there is no reason to believe that SIDS is not a problem in African populations as it has been wherever else SIDS has been studied. Prior systematic reviews have used data from studies performed in high-income countries [17]. To the best of our knowledge, no systematic review has focused on SIDS/SUID in Africa.

To fill this knowledge gap, we conducted a systematic review of SIDS/SUID studies conducted in Africa. Our review focused on two main questions:

1. What is the prevalence of known and hypothesized risk factors of SIDS/SUID in Africa?
2. What is the burden of SIDS and/or SUID deaths in Africa?

## METHODS

### Database and hand searching

PubMed, Embase, Web of Science, Cochrane, and Google Scholar were searched with search terms developed in collaboration with a librarian. The PubMed search was developed first using the following search terms: ((“Sudden Infant Death”[Mesh] OR “Sudden Infant Death Syndrome” OR “SIDS” OR “Sudden Infant Death” OR “Sudden Unexpected Infant Death” OR “Cot Death” OR “Cot Deaths” OR “Crib Death” OR “Crib Deaths” OR “Accidental Suffocation” OR “Unintentional Suffocation” OR “Strangulation in Bed” OR “ASSB”)) and an African search filter previously developed by Pienaar et al [18] (Appendix S1 in the **Online Supplementary Document**).

All articles up to December 26, 2020 (the date on which the search was executed) were included. There was no prior date restriction on the search. All returned articles were then imported into Covidence (Covidence.org, Melbourne, Australia, https://www.covidence.org), a systematic review software, for screening and data extraction. Duplicate articles were removed using the de-duplication feature in Covidence. Duplicate articles that were missed in this initial phase were removed during full-text review manually by the review authors.

### Selection process

Two review authors (GKO-P and ST) independently screened titles and abstracts to identify any relevant articles. Articles that passed this initial review were included in a full-text review. The full texts of included studies were then screened. Articles that met the inclusion criteria were included in the review. Disagreements were resolved through discussion between reviewers until consensus was reached.

### Inclusion/exclusion criteria

Articles were included for review if they met the following inclusion criteria:

- Original research articles, case reports, and case series were included. Editorials, letters to the editor, opinions, and review articles were excluded.
- Articles were restricted geographically to Africa (including North Africa and sub-Saharan Africa).
- Non-English language articles were included if an English translation was available or if it could be translated into English using Google Translate.
- Articles that specifically mentioned or reported data on Sudden Unexpected Infant Death (SUID) or Sudden Infant Death Syndrome (SIDS) (known or hypothesized risk factors or burden of disease) were included. Articles that reported on a population that included infants but were not necessarily limited to infants were included if they reported on the prevalence of SIDS/SUID in the cohort of infants.

### Data collection and analysis

We independently extracted the following data using customized data extraction forms in Covidence: author and institutional affiliation, source of funding and conflict of interest, year of publication, study design, study aim, country of the population studied, sample size including total number of infants studied, number and proportion of infants who died of SIDS and/or SUID, and reported prevalence of any risk factors.

We independently assessed risk of bias for each included study using the Newcastle Ottawa Scale for observational studies [19] or a modification [20-23]. Case-control and cohort studies were assessed on three main domains of selection, comparability, and ascertainment of exposure and outcome. For case reports/case series studies, we excluded items on comparability and adjustment since these studies were non-comparable [20-22]. We assessed cross-sectional/prevalence studies on representativeness of the sample and size, comparability between respondents in different outcome groups, and appropriateness and completeness of the statistical test/analysis [23].

We did not receive nor require ethical approval for this study, as it does not involve human and animal subjects.

**Prospero Registration Number:** CRD42021257261 (Protocol available here: https://www.crd.york.ac.uk/prospero/display_record.php?RecordID=257261)

## RESULTS

### Study characteristics

Our search yielded 880 articles. 221 were identified as duplicates and were removed by the de-duplication feature in Covidence. The titles and abstracts of 659 articles were then reviewed and 576 were judged to be irrelevant. For example, 67 studies were identified with the acronym SIDS which also refers to Small Island Developing States – these were deemed irrelevant.

**Figure 1:**
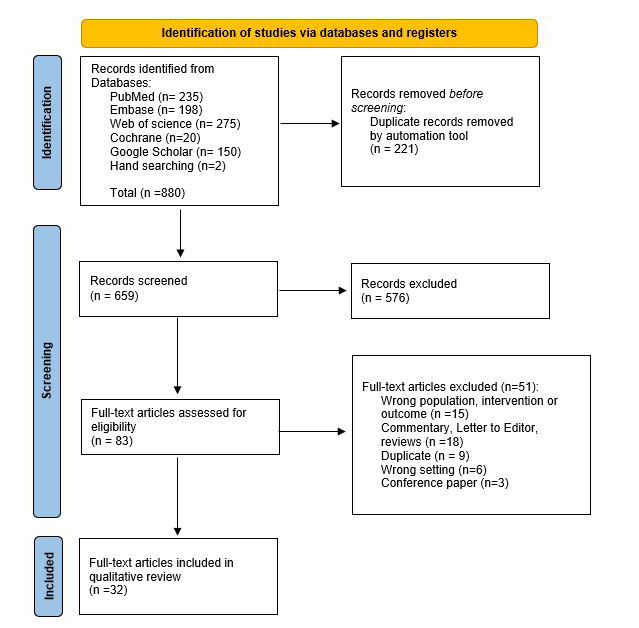
PRISMA flow diagram of search strategy. The PRISMA diagram details our search and selection process during the review. Source: [24]. For more information, visit: http://www.prisma-statement.org/.

The full texts of 83 articles were reviewed and 51 were excluded. These were: commentaries, editorials, and reviews (n = 18); studies that reported on the wrong population, exposure, or outcomes (n = 15); conference abstracts/papers (n = 3); wrong study setting or non-African studies (n = 6); and duplicate articles (n = 9) which were missed using the de-duplication feature in Covidence. Thirty-two full-text articles were included in the final qualitative review/synthesis (25 original studies and 7 case reports/case series). This process is summarized in the PRISMA flow diagram (**Figure 1**).

### Year of publication:

**Figure 2:**
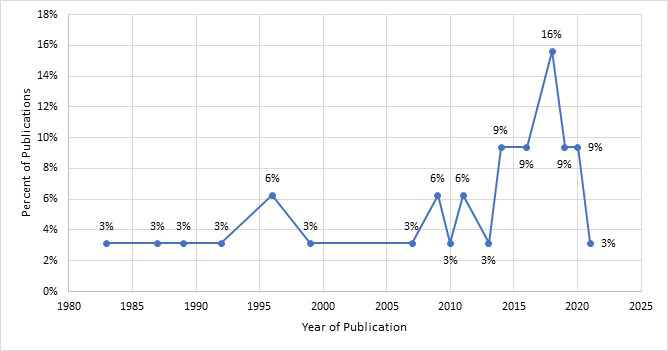
Distribution of included studies by year of publication. The figure shows the distribution of included studies by year of publication. Until recently, SIDS has been a low priority for researchers in Africa. More than half (52%) of the included studies were published between 2013 and 2021.

The included studies were published from 1983 to 2021 (we received the pre-print version of the 2021 article from the study authors in December 2020) with most conducted in the last decade. Sixty-nine percent were published between 2010 and 2021, with the majority in 2018 (n = 5, 16%) (**Figure 2****).**

### Geographic distribution of included studies

**Figure 3:**
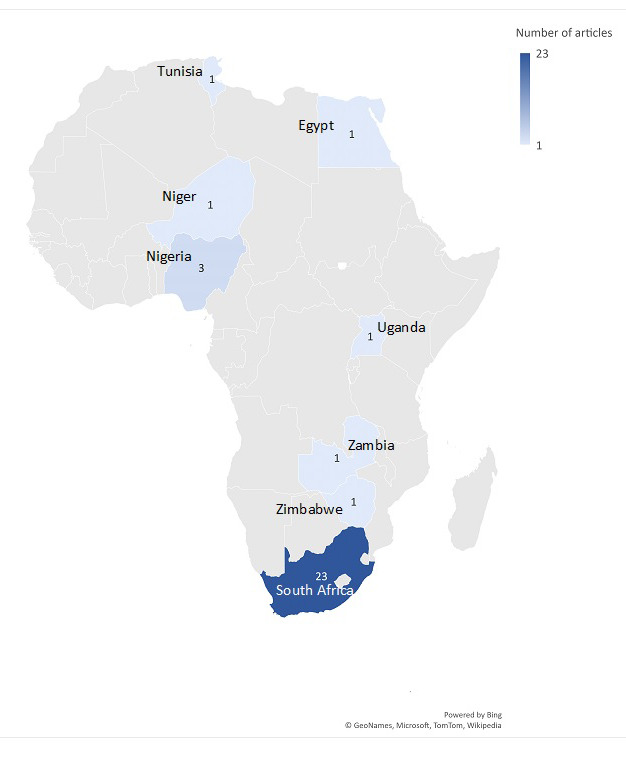
Distribution of included studies across the African continent. The figure shows the distribution of included studies across the African continent. Overall, the studies were skewed to southern Africa with the majority (72%) from South Africa. Slightly more than a quarter (28%) of the studies were conducted outside of South Africa.

Geographically, the articles were skewed to southern Africa (n = 25, 78%) with few from west Africa (n = 4, 13%), north Africa (6%), and east Africa (3%). The majority and nearly all of those from southern Africa were published in South Africa (n = 23, 72%) with three from Nigeria (9%) and one each from Egypt, Niger, Tunisia, Uganda, Zambia, and Zimbabwe (**Figure 3****)**.

### Quality scores

The 25 original articles were assessed for bias on a 9-point scale and classified as high (8-9), medium (5-7), or low quality (0-4). By study design, four case-control studies and three prospective cohort studies were included in this review. We rated all the case-control studies as medium quality, rated one cohort study as high quality and the remaining two as medium quality (**Table 1**). Eighteen studies were described as cross-sectional or retrospective/prospective audits. We rated the majority (67%, n = 12) of these studies as medium quality (**Table 2**).

**Table 1:** Methodological quality scores for case-control and prospective cohort studies*

Case control studies										
**Study, Year**	**Country**	**Case Definition**	**Representativeness of cases**	**Selection of controls**	**Definition of controls**	**Comparability of cases and controls**	**Ascertainment of exposure**	**Ascertainment of cases and controls**	**Non-response rate**	**Quality score**
Molteno, 1989 [25]	South Africa	1	1	1	1	0	1	1	1	7
Belonje, 1996 [26]	South Africa	1	0	1	0	0	1	1	1	5
Gaaloul, 2016 [27]	Tunisia	1	0	1	1	0	1	1	0	5
Van Deventer, 2018 [28]	South Africa	1	1	1	1	0	1	1	1	7
**Prospective cohort studies:**										
	**Country**	**Representativeness of cohort**	**Selection of non-exposed cohort**	**Ascertainment of exposure**	**Demonstration that outcome was not present at start of study**	**Comparability of cohorts**	**Ascertainment of outcome**	**Was follow-up long enough for outcomes to occur**	**Adequacy of follow-up of cohorts**	**Quality score**
Moyo, 2007 [29]	South Africa	1	0	1	1	0	1	1	1	6
Abdallah, 2018 [30]	Uganda	1	0	1	1	0	0	1	1	5
Elliott, 2020 [31]	South Africa	0	1	1	1	2	1	1	1	8

*A maximum of 2 points was assigned to comparability, all others were assigned a score of 1 if the criterion was satisfied; 0-4 was considered low quality; 5-7 was considered medium quality; and 8-9 was considered high quality.

**Table 2:** Methodological quality scores for cross-sectional/retrospective audits*

Study, year	Country	Representativeness of sample	Sample size	Non-respondents	Ascertainment of exposure	Comparability	Assessment of outcome	Statistical test	Quality Score
Vix, 1987 [32]	Niger	1	1	1	1	0	0	0	4
Potgieter, 1992 [33]	South Africa	1	0	0	1	0	1	1	4
Wolf, 1996 [34]	Zimbabwe	1	1	1	1	0	1	0	5
Kahn, 1999 [35]	South Africa	1	1	1	1	0	2	0	6
Ibeziako, 2009 [36]	Nigeria	1	1	1	1	2	2	1	9
duToit-Prinsloo, 2011 [37]	South Africa	1	1	1	0	0	1	0	4
duToit-Prinsloo, 2013 [38]	South Africa	1	1	1	1	0	2	0	6
Okpere, 2014 [39]	Nigeria	1	0	0	1	0	1	1	4
LaGrange, 2014 [40]	South Africa	1	1	1	1	0	1	1	6
Burger, 2014 [41]	South Africa	1	1	1	1	0	1	1	6
Reid, 2016 [42]	South Africa	1	1	1	1	0	2	1	7
Saayman, 2018 [43]	South Africa	1	0	0	1	1	1	1	5
Matshazi, 2018 [44]	South Africa	1	1	0	1	1	1	1	6
Elsobkey, 2018 [45]	Egypt	0	0	0	1	0	1	1	3
Ikenna, 2019 [46]	Nigeria	1	0	1	1	0	1	1	5
Bennett, 2019 [47]	South Africa	1	1	1	1	0	1	1	6
Heathfield, 2020 [48]	South Africa	1	1	1	1	0	2	1	7
Lapidot, 2021 [49]	Zambia	1	1	1	1	0	1	1	6

*A maximum of 2 points was allocated to comparability and assessment of outcome, all others were assigned 1 point if the criterion was met; 0-4 points was considered low quality; 5-7 was considered medium quality; and 8-9 was considered high quality.

The 7 case reports/case series were assessed for bias on a 5-point scale and rated similarly as high (5), medium (4), or low quality (0-3). Most of the case reports/case series were also rated as medium quality (57%, n = 4) as shown in **Table 3****.** Overall, 4 high-quality studies were included in this review.

**Table 3:** Methodological quality scores for case reports/case series studies*

Study, year	Country	Representativeness of case(s)	Diagnosis has been correctly made	Alternative diagnosis has been indicated	All important data has been cited	Outcome has been correctly ascertained	Quality score
Van Ieperen, 1983 [50]	South Africa	1	0	0	1	1	3
Randall, 2009 [51]	South Africa	0	1	1	1	1	4
Ker, 2010 [52]	South Africa	1	1	1	1	1	5
Dempers, 2011 [53]	South Africa	0	1	1	1	1	4
Dempers, 2016 [54]	South Africa	0	1	1	1	1	4
Heathfield, 2019 [55]	South Africa	1	1	1	0	1	4
Heathfield, 2020 [56]	South Africa	1	1	1	1	1	5

*Each criterion was assigned a score of 1; 0-3 was considered low quality; 4 was considered medium quality and 5 was considered high quality

### SIDS awareness

Awareness of SIDS was low in the general population. Three studies provided data on SIDS awareness, reporting a SIDS awareness rate of between 12.2% to 44.3% [39,45,46]. Two of these studies were of low quality and one of medium quality. The medium-quality study reported that 49 (12.2%) of respondents claimed to have heard of SIDS but only 5 (1.2%) had good knowledge of SIDS in a survey of 401 mothers of infants in Enugu, Nigeria [46] (**Table 4**).

**Table 4:** Characteristics and findings of studies focused on infant sleep practices and other maternal risk factors

Study, year	Country	Study design	Sample size and population studied	Prone position	Side position	Supine position	Bedsharing	Other relevant findings
Potgieter, 1992 [33]	South Africa	Cross-sectional study	416 mothers with infants aged 6 d to 6 mo	63.8%	33.5%	2.7%	60.0% (94.0% black, 71.0% colored, 4.0% white)	
Ibeziako, 2009 [36]	Nigeria	Cross-sectional study	480 mothers with infants aged 1 to 52 weeks	26.7%	51.8%	21.5%	66.9%	
Okpere, 2014 [39]	Nigeria	Cross-sectional study	282 mothers of infants aged less than 1 y who presented to well-baby clinics	44.3%	20.6%	18.1%	63.7% with mother (33.5% with both parents; 2.8% with other child)	SIDS awareness, 35.1%
								81.2% were unaware of recommended sleep position for infants
Burger, 2014 [41]	South Africa	Postmortem retrospective case audit	82 deceased infants admitted as SUID cases	24.0%			65.0%	Prematurity, 27.0%
								Parental smoking, 29.0%
								Parental alcohol use, 24.0%
LaGrange, 2014 [40]†	South Africa	Postmortem prospective descriptive study	148 deceased infants presenting as SUID cases at Tygerberg Medico-Legal Mortuary	30.5% (32/105)			69.5% (73/105)	Peak age of SUID, 1-2months
								Male vs female (60.1% vs 39.9%)
								Wrapped in thick heavy blankets, 51.4% (54/105)
								Parental smoking, 39.0% (41/105)
								Parental alcohol use, 37.1% (39/105)
Elsobkey*, 2018 [45]	Egypt	Quasi-experimental study	70 mothers of preterm neonates with gestational age between 32 and 37 weeks, and weighing >1500 g			22.9%		SIDS awareness, 44.3% (Classified as average to good knowledge of SIDS)
								Firm sleep surface, 40.0%
								Avoid smoke exposure, 22.9%
Saayman, 2018 [43]†	South Africa	Postmortem descriptive study	168 deceased infants presenting as SUID cases at Tygerberg Medico-Legal Mortuary	23.6% (33/140)	64.3% (90/140)	12.1% (17/140)	96.0% (144/150)	Prenatal alcohol, 18.0% (29/161)
								Prenatal tobacco smoke exposure, 31.0% (50/161),
								Postnatal tobacco smoke exposure, 11.0% (15/136)
Matshazi, 2018 [44]†	South Africa	Postmortem descriptive study	183 deceased infants aged less than 1 y admitted as SUID cases to Tygerberg Medico-Legal Mortuary	37.0% (37/101)	53.0% (54/101)	10.0%	95.0% (96/101)	Tobacco smoke exposure, 31.0%,
								Prenatal alcohol use, 20.0%
Ikenna, 2019 [46]	Nigeria	Cross-sectional study	401 mothers of infants aged less than 1 y	29.4%	45.9%	11.7%	91.8%	SIDS awareness, 12.2%
								Tobacco smoke exposure, 10.2%
								Incidence of SUID, 7.7% (described as mothers who witnessed sudden infant death)
Heathfield, 2020 [48]	South Africa	Postmortem retrospective case audit	1199 deceased infants admitted as SUID cases to Salt River Mortuary				94.7%	Previous history of SUID, 12.8%
								Peak age of SUID, 1-2 mo
								Male vs female (51.7% vs 48.3%)
								Prematurity, 40.6%
								Tobacco smoke exposure, 53.1%
								Maternal alcohol use, 19.8%

y – years, mo – months

*This was a pre/post study. Prevalence estimates are baseline results

†Thesis/Dissertation study.

### Risk factors for SIDS

#### Risk factors intrinsic to the infant

The peak age of SIDS/SUID varied across studies. Two medium quality South African studies found the peak age of SIDS/SUID deaths to be 1-2 months [40,48] while another reported a peak age of 2-4 months [37]. Slightly more male than female infants died of SIDS/SUID [37,40,48]. Three medium-quality studies, two from South Africa and the other from Uganda, explored prematurity as a risk factor of SIDS. The South African studies reported that 27% to 40.6% of SUID cases were preterm [41,48]. The Ugandan study found that suspected SIDS was the second leading cause of death in a cohort of preterm infants, accounting for nearly 25% of all deaths in that cohort [30].

#### Risk factors intrinsic to the mother

Six studies provided prevalence estimates on maternal/parental smoking and alcohol use. One medium-quality study reported a 10.2% prevalence of exposure to tobacco smoke in Nigeria among mothers at a well-baby clinic [46]. Among SUID cases, exposure to tobacco smoke was reported at a rate of 29% to 53.1% in South Africa [40,41,43,44,48]. Maternal use of alcohol was reported at a rate of 18% to 37.1% among SUID cases in South Africa [40,41,43,44,48]. One high-quality study in South Africa specifically focused on estimating the risk of SIDS in a cohort of infants due to prenatal exposure to alcohol and tobacco smoke. The study found that the adjusted relative risk for SIDS was 2.6 times higher for those who were exposed to alcohol compared to those who were not, and 3.8 times higher for those who were exposed to smoking compared to those who were not [31]. The study cohort included mothers and infants from South Africa and the US [31] It is not clear what the actual risk is in the African cohort.

#### Infant sleep practices

Nine of the included studies reported on infant sleep practices in Nigeria, Egypt and South Africa, either as the main outcome of the study or as secondary outcomes [33,36,39,41,43-46,48]. Four articles reported on infant sleeping practices among mothers of infants at well-baby clinics while the remainder of these articles reported on infant sleep practices among SUID cases admitted to medico-legal laboratories in South Africa. The prone sleeping position was preferred by 26.7% to 63.8% of mothers of infants at well-baby clinics [33,36,39,46]. The lateral sleep position was preferred by 20.6% to 51.8% of mothers [33,36,39,46]. Pactice of the recommended supine position for infants is less common. A minority (2.7% to 21.5%) of mothers placed their babies in the supine position during sleep [33,36,39,46].

Among SUID cases, a majority of infants were reported to have been placed in the side position prior to death. The proportion of SUID cases placed to sleep in the side position was reported as 53% to 64% compared to 10% to 12% in the supine or back position [43,44]. 23.6% to 37% of SUID cases were placed in the prone position [41,43,44]. Bedsharing was also very common. Bedsharing was reported at a rate of 60% to 91.8% among mothers of infants at well-baby clinics [33,36,39,46]. Among SUID cases, the rate was nearly 95% [48]. One included study using post-mortem biomarkers of hypoxia did not find any significant differences between hypoxanthine and urate concentrations in vitreous humor samples of SIDS victims and infants who died of other causes [26]. **Table 4** summarizes the main findings of studies focused on infant and maternal risk factors.

#### Infectious risk factors

Six studies explored the role of infectious agents in the pathogenesis of presumed SIDS in Africa. One medium-quality study highlighted the likely role of tuberculosis in SIDS-like deaths and found evidence of primary pulmonary tuberculosis on autopsy in a 4.5-month-old male infant whose history and death scene investigation fit the profile of a SIDS death [53]. The remaining five studies explored the role of viruses in SIDS deaths. These medium-quality studies used PCR testing to detect viral pathogens in a cohort of SIDS/SUID infants. The commonest viruses detected were HRV, RSV, HCoV, and CMV [40,41,43,44]. Viruses were detected in nearly half of the SIDS cases using PCR in South Africa [40]. Another medium-quality study detected Coxsackie B virus in nearly 23% of presumed SIDS cases in Tunisia [27] (**Table 5**).

**Table 5:** Characteristics and findings of studies that assessed the role of infections and genetic factors in SIDS/SUID

Study, year	Country	Study design	Sample size and population studied	Significant factor	Relevant findings
Dempers, 2011 [53]	South Africa	Case report study	1 deceased male infant aged 4.5 mo who died suddenly and unexpectedly at a day care	Primary TB	Postmortem findings were consistent with rogressive primary pulmonary TB
LaGrange, 2014 [40]†	South Africa	Postmortem prospective descriptive study	148 deceased infants presenting as SUID cases at Tygerberg Medico-Legal Mortuary	Respiratory viruses in SUID cases (HRV, RSV, HCoV, Human enterovirus, HMPV, Influenza A&B)	PCR positive HRV in 68 (46.0%), RSV A&B in 16 (10.8%), HCoV in 12 (8.1%), Human enterovirus in 6 (4.1%), HMPV in 5 (3.4%), PIV3 in 4 (2.7%) and Influenza A&B in 4 (2.7%)
					(PCR positive viruses in 50.0% of SIDS cases, 74.5% in deaths classified as infection and 37.5% in deaths classified as Other)
					SIDS diagnosed in 33.7% (34/101)
Burger, 2014 [41]	South Africa	Postmortem retrospective case audit	82 deceased infants admitted as SUID cases	Adenovirus, CMV and RSV	PCR positive Adenovirus in 2 (2%), and cytomegalovirus in 29 (35%).
					RSV detected in 4 (5%) cases using IHC
Gaaloul, 2016 [27]	Tunisia	Case-control study	56 deceased infants aged 2 to 11 mo (39 SIDS cases and 17 unnatural home death controls)	Coxsackie B virus	PCR positive Coxsackie B virus in 9 SIDS cases (23.0%)
					(Enterovirus detected by IHC and PCR in 6 SIDS cases (15.3%) with myocarditis and 3 (7.7%) with peri myocarditis)
Saayman, 2018 [43]†	South Africa	Postmortem descriptive study (cross-sectional)	168 deceased infants presenting as SUID cases at Tygerberg Medico-Legal Mortuary	EV and B19	PCR positive EV and B19 in 49 cases (29%)
					SIDS diagnosed in 40% (48/121)
Matshazi, 2018 [44]†	South Africa	Postmortem descriptive study (cross-sectional)	183 deceased infants aged less than 1 y admitted as SUID cases to Tygerberg Medico-Legal Mortuary	Respiratory viruses in SUID cases	PCR positive Human Rhinovirus A/B/C in 65 (35.5%), Adenovirus in 18 (12.6%), Parainfluenza 3 in 10 (6.0%), Enterovirus in 9 (4.9%) and RSV B in 7 (3.8%) cases
					SIDS diagnosed in 48.3% (57/118)
Van Ieperen, 1983 [50]	South Africa	Case report study	3 male siblings aged 6, 3, and 7 weeks who died suddenly and unexpectedly at home during sleep	Genetic etiology: Sibling history	Postmortem findings showed possible genetic abnormality in second case and rapid hypoxia probably caused by smothering in third case. First case was ruled as a natural death since no postmortem was performed
Ker, 2010 [52]	South Africa	Case report study	1 deceased male infant aged 3 mo who presented with SIDS	Genetic etiology: Cardiac disorders – Left ventricular hyper trabeculation	Postmortem findings concluded that death was due to fatal arrhythmia from left ventricular hyper trabeculation (Postmortem revealed numerous apical trabeculations of left ventricle)
vanDeventer, 2018 [28]	South Africa	Retrospective case audit (genetic study)	48 FFPE tissue samples from SUID cases, 10 control FFPE samples from deceased infants with known cause of death and 9 blood samples from healthy volunteers	Genetic etiology: SCN5A	Pathogenic/probably pathogenic genetic variants detected in 10 cases (20.8%)
					SCN5A variants associated with LQTS was detected in 6.2% of cases (3/48)
Heathfield, 2019 [55]	South Africa	Case report study	1 deceased male infant* aged 2 mo admitted as SUID	Genetic etiology: SCN10A	Rare putatively pathogenic variant was found in SCN10A gene (SCN10A is linked to Brugada syndrome)
					(Infant was homozygous for this rare variant)
Heathfield, 2020 [56]	South Africa	Case report study	1 deceased female infant* aged 3 mo	Genetic etiology: GALT: c.404C>G	Genetic testing found that infant was homozygous for GALT: c.404C>G
					(Estimated prevalence: 1 infant out of 102 black African SUID cases)

y – years, mo – months

HCoV – human coronavirus, HRV - human rhinovirus, EV – enterovirus, CMV – cytomegalovirus, RSV – respiratory syncytial virus, B19 – parvovirus B19, HMPV – human metapneumovirus, PIV3 – parainfluenza virus type 3, IHC – Immunohistochemistry, PCR – polymerase chain reaction, FFPE – formalin fixed, paraffin-embedded (FFPE), SCN5A – sodium voltage-gated channel alpha subunit 5, SCN10A – sodium voltage-gated channel alpha subunit 10, LQTS – long QT syndrome, GALT – galactose-1-phosphate uridylyl transferase, TB – tuberculosis

*Infant was of African ancestry.

†Thesis/Dissertation study.

#### Genetic risk factors

Five studies, all from South Africa, explored the role of genetic risk factors in SIDS/SUID cases. The earliest study is a case report from 1983 which found genetic factors to be the likely cause of death in one case of three sudden infant deaths in the same family [50]. Recently, one medium-quality study found that 12.8% of mothers of SUID cases had a previous history of SUID [48] (**Table 2**). Pathogenic/probably pathogenic genetic variants were detected in two of these studies. One medium-quality study detected pathogenic/probably pathogenic genetic variants in 20.8% of the SUID cases studied [28]. The *SCN5A* variant which is associated with the long QT syndrome was detected in 6.25% of cases [28]. Another case report detected a pathogenic variant in the *SCN10A* gene, a gene associated with Brugada syndrome, in a three-month-old male infant who had died of SUID [55]. Other genes and anatomic abnormalities identified in these African studies included GALT:c.404c>G, a gene associated with galactosemia [56], and left ventricular hyper-trabeculation (an anatomic defect that can lead to fatal arrhythmias) [52] (**Table 5****).**

### Burden of SIDS/SUID

Eleven studies explored the burden of SIDS/SUID in Africa. These studies provided very wide-ranging rates of SIDS in Africa, from an implausibly low rate of 0.2 per 1000 live births as reported from a study in Zimbabwe [34] to a high of 3.89 per 1000 live births in South Africa [25]. The Zimbabwean study estimated a SIDS prevalence rate of 0.2 per 1000 live births in the general population. However, we rated their statistical analyses to have a high risk of bias since the denominator for the population at risk was not the same from which the infants with apparent SIDS were sampled. In addition, one study from Niger reported a SIDS prevalence rate of 2.5 per 1000 live births in healthy infants and 40 per 1000 live births in infants with sickle cell disease [32]. We also rated this study as low quality since there was a high risk of bias in the statistical analysis.

The South African studies provided relatively stronger estimates of the SIDS prevalence rate in the general population. The earliest estimate of SIDS prevalence in South Africa was in 1989 when one medium-quality study reported a SIDS prevalence rate of 3.01 per 1000 live births [25]. Recently, one high-quality prospective cohort study reported an unadjusted risk of SIDS in a cohort of infants in Cape Town as 3.7 per 1000 live births [31]. Among deceased infants, SIDS accounted for between 2.5% to 21% of infant deaths in South Africa [37,38,42,54]. However, very few studies outside of South Africa provided estimates on the proportion of infant deaths due to SIDS. One medium-quality study from Zambia estimated that 11.3% of infant deaths were due to suspected SIDS [49] (**Table 6****)**.

**Table 6:** Characteristics and findings of studies on the burden of SIDS/SUID and diagnostic challenges in Africa

Study, year	Country	Study design	Sample size and population studied	Significant factor	Relevant findings
Vix, 1987 [32]	Niger	Cross-sectional study	400 mothers of infants at well-baby clinics	SIDS	SIDS prevalence per 1000 live births: 2.5 in healthy infants. 40 in sickle cell infants
Molteno, 1989 [25]	South Africa	Case-control study	299 children aged 1 mo to 5 y (199 cases and 100 healthy controls)	SIDS, other causes of early childhood death: deaths determined at birth and deaths from accidents and acquired disease	SIDS incidence per 1000 live births: 3.89 overall, 3.05 if obvious cause of death is removed at autopsy (White 1.05 and Colored 3.41)
Wolf, 1996 [34]	Zimbabwe	Postmortem prospective descriptive study	180 deceased infants aged 1 mo to 1 y who died at home	SIDS	SIDS incidence per 1000 live births: 0.20 (95% CI: 0.004 - 0.4) [4 cases out of 18 889 live births]
Kahn, 1999 [35]	South Africa	Cross-sectional study (Demographic and health surveillance)	216 children under 5 y	SIDS	2 SIDS deaths (Number of infants aged <1 y is unclear)
Moyo, 2007 [29]	South Africa	Prospective cohort study	11 677 children enrolled in a Tuberculosis vaccine field trial	SUID	SUID prevalence per 1000 live births: 1.03 per 1000
					SUID prevalence among deceased infants 8.2% (12/146)
duToit-Prinsloo, 2011 [37]	South Africa	Retrospective case audit	813 deceased infants younger than 1 y of age that were admitted to the medico-legal mortuaries of Pretoria and Tygerberg	SIDS	SIDS prevalence among deceased infants 21.0% (171/813)
duToit-Prinsloo, 2013 [38]	South Africa	Retrospective case audit	2583 deceased infants younger than 1 y of age that were admitted to 5 academic medico-legal centers across 4 provinces in South Africa	SUID	SIDS prevalence among deceased infants 8.7% (224/2583)
Reid, 2016 [42]	South Africa	Retrospective case audit	700 deceased children aged less than 5 y in the Metro West geographical area of the Western Cape Province in South Africa	Under-5 mortality	SIDS prevalence among deceased infants 2.5% (14/564)
Dempers, 2016 [54]	South Africa	Case series	18 deceased infants admitted as SUID cases	SIDS	SIDS prevalence among deceased infants 38% (7/18) based on 1990 NICHD schema
Abdallah, 2018 [30]	Uganda	Prospective cohort study	164 preterm infants with birth weight less than or equal to 1500g	Cause of mortality in preterm infants	Suspected cot death 4.9% (8/164)
					SIDS prevalence among deceased infants 25.0% (8/32)
Elliott, 2020 [31]	South Africa	Prospective cohort study	10 088 pregnant women in two residential areas within Cape Town South Africa and five areas in the United States; 6240 infants from the South African site	SIDS	SIDS incidence per 1000 live births: 3.70 per 1000 live births (unadjusted)
					Adjusted relative risk of SIDS: Alcohol 2.59 (95% CI = 1.14-5.90, *P* = 0.024); Smoking 3.84 (95% CI = 1.42-10.42, *P* = 0.008) (Continuous/quit late vs None/quit early)
Lapidot, 2021 [49]	Zambia	Postmortem prospective descriptive study	230 deceased infants aged 4 d to 6 mo	SUID	SUID prevalence among deceased infants 11.3% (26/230)
Belonje, 1996 [26]	South Africa	Case-control study	84 infants aged less than 1 y (50 SIDS cases and 34 controls who died of other causes	Hypoxanthine and Urate as biomarkers of SIDS	No difference in hypoxanthine concentration between SIDS victims and other causes of death (*P* value of difference in mean concentration of Hypoxanthine at 1, 2, 3, 4 and 5 postmortem interval days is 0.862, 0.014, 0.331, 0.424 and 0.508 respectively)
Randall, 2009 [51]	South Africa	Case series study	10 deceased infants, median age 2 mo admitted as SUID cases	Classification schema	SIDS was diagnosed in 6 infants using standard classification schema compared to 2 infants using new classification schema
Bennett, 2019 [47]	South Africa	Retrospective case audit	454 deceased infants admitted as SUID cases	Death scene investigation practices	Proportion of SUID cases with death scene investigation 59.2%
					Proportion of infant deaths due to SUID 6.6% (454/6922)

y – years, mo – months

### Diagnostic challenges

Two studies reported on the challenge of making a diagnosis of SIDS. One medium-quality study from South Africa reported on the inadequacy of death scene investigation in SUID cases in South Africa. They noted that only 59.2% of SUID cases had a complete death scene investigation [47]. To account for the uncertainty posed by an asphyxia risk in making an accurate diagnosis of SIDS, study authors in another medium-quality study incorporated asphyxia in a new classification schema for SUID cases. They found that this classification schema performed well in assigning the cause of death compared to the standard classification schema [51] (**Table 6**).

## DISCUSSION

Our main conclusions are that, with the singular exception of studies from South Africa, there is a paucity of information about the risk factors for or burden of SIDS in Africa. Overall, this supports our initial concerns that SIDS in Africa has historically been a very low priority for the global health community, except for a recent set of publications. And yet there is no reason to believe that SIDS would not be a major cause of infant mortality in Africa as it has proven to be wherever else SIDS has been studied. In support of this argument, our review found a high burden of SIDS/SUID and high rates of known risk factors of SIDS in Africa. The rates of the prone and side sleeping positions in this review are much higher than the rates reported from other countries such as the US and the UK. In the UK, the prone sleeping position has remained relatively stable at a rate of 23% to 24% in recent years [57]. In the US, 7.8% of mothers reported placing their infants to sleep in the prone position in a study of 3297 mothers [58]. Additionally, the CDC reported that 21.9% of mothers placed their infants to sleep in a non-supine position in 2015 [59]. In Brazil, findings from the 2015 Pelotas Birth Cohort study estimated that less than 2% of mothers placed their infants in the prone sleeping position [60]. The American Academy of Pediatrics (AAP) recommends that infants be placed in the supine position to sleep. The AAP further recommends that infants do not share the same sleep surface with their caregivers [5]. It is worrying that very few infants are placed in the recommended supine/back position to sleep in this review. The reported rates of 2.7% to 21.5% are much lower than the rates reported elsewhere (77% in the US) [58]. The side or prone sleeping position poses a risk of rebreathing expired gases which can lead to hypoxia or hypercapnia [7]. The results of one included study did not support the view that pre-mortem hypoxia is a common feature in SIDS when compared with other causes of death [26]. However, the validity of this forensic tool in the evaluation of SIDS has recently been called into question [61].

The rates of bedsharing of 60% to 91.8% in this review are also much higher than the rates reported from the US and Australia. In the US, it is estimated that 20.7% to 24.4% of mothers reported bedsharing with their infants [59,62]. In Australia, a study by Cunningham et al. revealed a 44.7% bedsharing rate among 2745 mothers in Victoria [63]. Since bedsharing and prone or side sleeping appear to be highly prevalent in the African studies in our review, there appears to be a significant unexplored opportunity to reduce infant mortality in these settings.

Previous studies have established prematurity as a risk factor of SIDS [64-66]. Findings from this review suggest a high risk of SIDS for preterm infants in Africa. Almost half of the SUID cases in South Africa were preterm. Moreover, SIDS was the leading cause of death among a cohort of preterm infants in Uganda. These findings are consistent with results from developed countries. Malloy in 2013 showed that despite the decline in SIDS rates among term infants, the risk of SIDS among the preterm remained high [66]. We also found high rates of maternal smoking and alcohol use among mothers of infants with SUID in South Africa. For instance, almost half of the SUID cases in South Africa were exposed to tobacco smoke either through the mother or another person in the household, and more than a quarter of these mothers reported using alcohol [48]. The reported rates of tobacco smoke exposure to infants in this review are also higher than the rates reported elsewhere. Using linked birth and infant death data from 2007 to 2011, one large study in the US reported that 8.9% of mothers smoked during pregnancy [67] compared to the 10.2% found in this review [46].

Infectious agents and genetic factors have been suggested as likely causes in the pathogenesis of SIDS [8,9]. There is evidence to suggest that viral agents play a role in the pathogenesis of SIDS either directly or indirectly through interactions with bacteria [68]. Previous studies have suggested that 80% of SIDS cases report a mild upper respiratory tract infection in the days prior to death [8,68]. Respiratory viruses were detected in nearly half of the SIDS/SUID cases in this review, lending credence to the possible role of respiratory viruses in the pathogenesis of SIDS. In addition, genetic testing detected pathogenic/probably pathogenic genetic variants in nearly 21% of SUID cases in one included study and a pathogenic variant of the *SCN5A* gene in 6.25% of SIDS cases in another included study in this review. Our findings are consistent with prior research by Weese-Mayer et al. who estimated that between 5% to 10% of SIDS cases had novel mutations in the *SCN5A* gene leading to the long QT syndrome [69]. These studies confirm the need for more detailed investigations to fully identify the cause of death in SIDS/SUID cases. Given the low rates of genetic testing in Africa, these causes of infant mortality are likely going undetected. Whether this represents another opportunity to reduce infant mortality in Africa is very unclear, however. Prospective screening has failed to be effective in high-income settings, making it hard to argue for operationalizing this ineffective strategy in a low-resource setting. They however highlight that these lesser-known risk factors of SIDS are likely present in Africa.

Findings from this review also indicate that Africa likely has some of the highest rates of SIDS in the world. Relying on methodological quality, the most recent estimate from South Africa indicates a SIDS rate of 3.7 per 1000 live births [31]. This rate is significantly higher than current estimates from the UK (0.3 per 1000 live births)[57], US (0.3 per 1000)[70], Australia (SUID rate 0.5 per 1000), Germany (0.53 SUID rate per 1000) and Japan (0.6 SUID rate per 1000) [6]. Collectively these studies suggest that SIDS probably accounts for a larger share of infant deaths in Africa than has generally been appreciated. Given the high rates of prone/lateral sleeping position and bedsharing in this review, more studies conducted outside of South Africa may find the SIDS burden across Africa is actually even higher.

Ultimately, SIDS is a diagnosis of exclusion and can only be diagnosed when other causes of death have been ruled out following death scene and detailed post-mortem investigations. Most countries in Africa lack the resources to conduct a proper SIDS investigation. Even South Africa, which is sort of a pioneer in SIDS investigations, lags other well-developed economies. Moreover, distinguishing between SIDS and suffocation deaths due to an unsafe sleep environment can be challenging. This challenge is emphasized when one considers that infants who may have a genetic predisposition to SIDS may only experience SIDS in the setting of an additional proximal factor, such as sleep position, bedding composition, or swaddling practices.

### Strengths and limitations

The main strength of this review is that this is the first systematic review on SIDS in Africa. To our knowledge, no other review has been conducted on SIDS/SUID using studies from Africa. Our study is not without limitations. The majority of the included studies were conducted in South Africa which may affect the generalizability of our findings to the entire continent. However, most of the South African studies were conducted on predominantly Black or bi-racial populations and thus results can be extrapolated to other similar populations on the continent.

## CONCLUSIONS

National campaigns to promote a safe sleep environment are lacking in Africa. The “back to sleep” campaign in the UK for instance led to a 40% decline in the SIDS rate in the first year alone [57]. Similar declines were noted in the US following the implementation of safe sleep campaigns [71]. These campaigns target some of the major risk factors of SIDS, such as prone sleeping and bedsharing [57], and would be worthwhile in Africa to tackle the high infant mortality rates. However, the paucity of high-quality studies outside of South Africa limits our ability to make recommendations for such campaigns. Future research should focus on prospectively estimating the prevalence of SIDS in countries other than South Africa.

## Additional material


Online Supplementary Document
