# Supplementary material for: A systematic review of the burden and risk factors of sudden infant death syndrome (SIDS) in Africa
Source: J Glob Health. 2021 Dec 25;11:04075. doi: 10.7189/jogh.11.04075 (PMC8719309; doi:10.7189/jogh.11.04075)
Supplement: Online Supplementary Document [file jogh-11-04075-s001.pdf]

Appendix S1. Full PubMed Search Filter:

SIDS/SUID in Africa Search Filter

(("Sudden Infant Death"[Mesh] OR "Sudden Infant Death Syndrome" OR "SIDS" OR "Sudden
Infant Death" OR "Sudden Unexpected Infant Death" OR "Cot Death" OR "Cot Deaths" OR "Crib Death" OR "Crib Deaths" OR "Accidental Suffocation" OR "Unintentional Suffocation"
OR "Strangulation in Bed" OR "ASSB")) AND ((‘‘Africa’’[MeSH] OR Africa*[tw] OR Algeria[tw] OR Angola[tw] OR Benin[tw] OR Botswana[tw] OR ‘‘Burkina Faso’’[tw] OR Burundi[tw] OR Cameroon[tw] OR ‘‘Canary Islands’’[tw] OR ‘‘Cape Verde’’[tw] OR ‘‘Central
African Republic’’[tw] OR Chad[tw] OR Comoros[tw] OR Congo[tw] OR ‘‘Democratic Republic of Congo’’[tw] OR Djibouti[tw] OR Egypt[tw] OR ‘‘Equatorial Guinea’’[tw] OR Eritrea[tw] OR Ethiopia[tw] OR Gabon[tw] OR Gambia[tw] OR Ghana[tw] OR Guinea[tw] OR
‘‘Guinea Bissau’’[tw] OR ‘‘Ivory Coast’’[tw] OR ‘‘Cote d’Ivoire’’[tw] OR Jamahiriya[tw] OR
Kenya[tw] OR Lesotho[tw] OR Liberia[tw] OR Libya[tw] OR Libia[tw] OR Madagascar[tw] OR Malawi[tw] OR Mali[tw] OR Mauritania[tw] OR Mauritius[tw] OR Morocco[tw] OR Mozambique[tw] OR Mocambique[tw] OR Namibia[tw] OR Niger[tw] OR Nigeria[tw] OR Principe[tw] OR Reunion[tw] OR Rwanda[tw] OR ‘‘Sao Tome’’[tw] OR Senegal[tw] OR Seychelles[tw] OR ‘‘Sierra Leone’’[tw] OR Somalia[tw] OR ‘‘South Africa’’[tw] OR ‘‘St Helena’’[tw] OR Sudan[tw] OR Swaziland[tw] OR Tanzania[tw] OR Togo[tw] OR Tunisia[tw]
OR Uganda[tw] OR ‘‘Western Sahara’’[tw] OR Zaire[tw] OR Zambia[tw] OR Zimbabwe[tw]
OR ‘‘Central Africa’’[tw] OR ‘‘Central African’’[tw] OR ‘‘West Africa’’[tw] OR ‘‘West African’’[tw] OR ‘‘Western Africa’’[tw] OR ‘‘Western African’’[tw] OR ‘‘East Africa’’[tw] OR
‘‘EastAfrican’’[tw] OR ‘‘Eastern Africa’’[tw] OR ‘‘Eastern African’’[tw] OR ‘‘North Africa’’[tw] OR ‘‘North African’’[tw] OR ‘‘Northern Africa’’[tw] OR ‘‘Northern African’’[tw]
OR ‘‘South African’’[tw] OR ‘‘Southern Africa’’[tw] OR ‘‘Southern African’’[tw] OR ‘‘sub Saharan Africa’’[tw] OR ‘‘sub Saharan African’’[tw] OR ‘‘subSaharan Africa’’[tw] OR ‘‘subSaharan African’’[tw]) NOT (‘‘guinea pig’’[tw] OR ‘‘guinea pigs’’[tw] OR ‘‘aspergillus
niger’’[tw]))

NB: The PubMed search filter was adapted to the other databases with minor modification
